# Supplementary material for: White matter disconnectivity fingerprints causally linked to dissociated forms of alexia
Source: Commun Biol. 2021 Dec 20;4:1413. doi: 10.1038/s42003-021-02943-z (PMC8688436; doi:10.1038/s42003-021-02943-z)
Supplement: Supplementary file 2 — Supplementary Information [file 42003_2021_2943_MOESM2_ESM.pdf]

Supplementary information

White matter disconnectivity fingerprints causally linked to dissociated forms of alexia

Sam Ng,[1,2*] Sylvie Moritz-Gasser,[1,2,3] Anne-Laure Lemaitre,[1,2] Hugues Duffau[1,2] and Guillaume Herbet[1,2,3]

[1]Department of Neurosurgery, Gui de Chauliac Hospital, Montpellier University Medical Center, Montpellier, France

[2]Institut de Génomique Fonctionnelle, Université de Montpellier, CNRS, INSERM, Montpellier, France

[3]Department of Speech-Language Pathology, University of Montpellier, Montpellier, France

*Corresponding author:

Sam Ng, MD

s-ng@chu-montpellier.fr

Summary (order in which they appear in the main text):

Supplementary Figure 1: Spatial distribution of subcortical stimulation sites (n=72).

Supplementary Table 1: Montreal Neurological Institute coordinates (reading disorders)

Supplementary Table 2: Montreal Neurological Institute coordinates (speech disorders)

Supplementary Figure 2: Tract-level analyses. Distribution of "disconnected" streamlines by VOI size.

Supplementary Figure 3: Tract-level analyses. Distribution of "disconnected" streamlines by angular threshold.

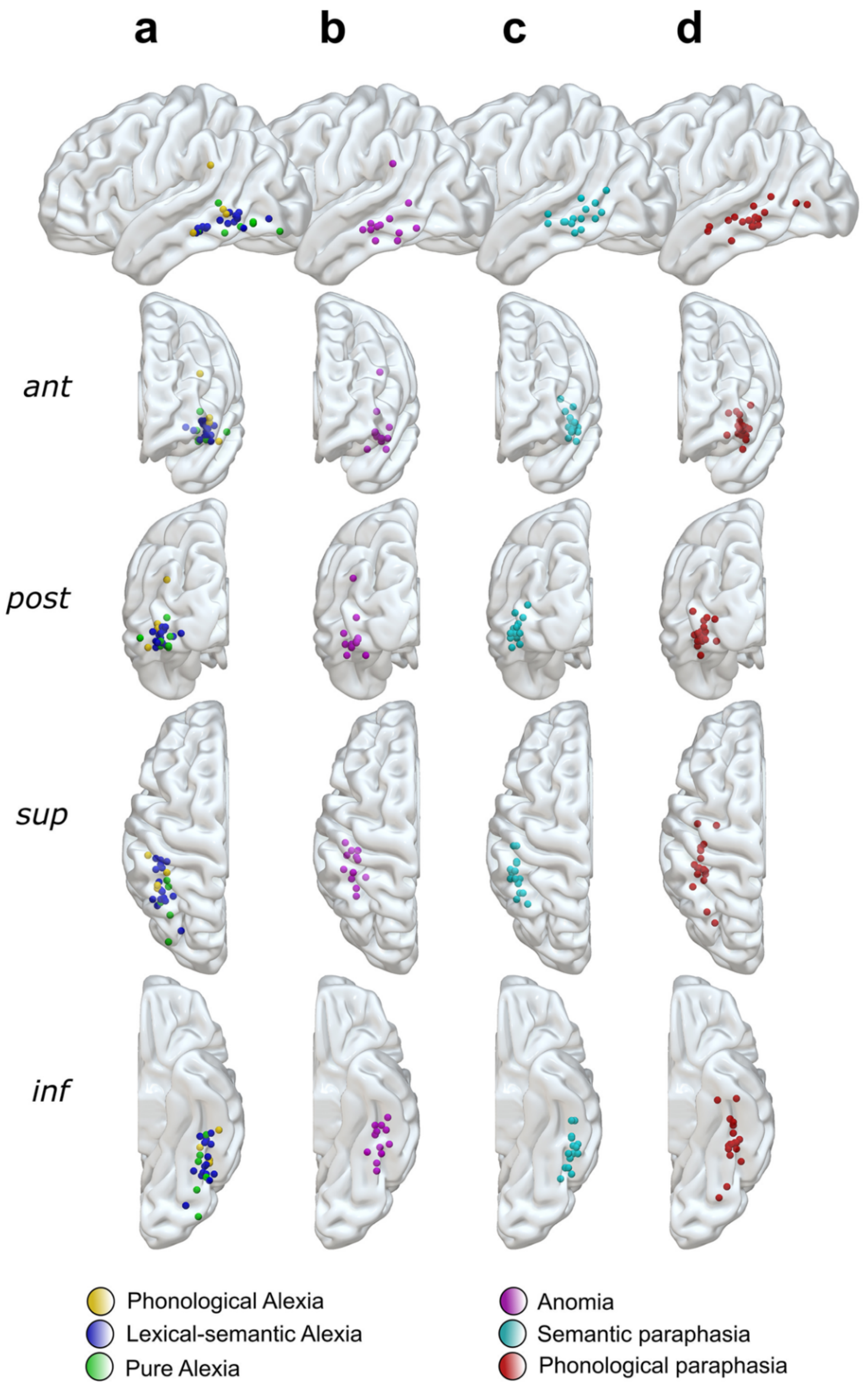

Supplementary Figure 1: Spatial distribution of subcortical stimulation sites (n=72). (**a**) Stimulations eliciting alexia (n=27). (**b**) Stimulations eliciting anomia (n=12). (**c**) Stimulations eliciting semantic paraphasia (n=18). (**d**) Stimulations eliciting phonological paraphasia (n=15).

Supplementary Table 1: Montreal Neurological Institute (MNI) coordinates (reading disorders)

Stimulations	X coordinate	Y coordinate	Z coordinate
**Ph-A**			
S1	-46	-48	0
S2	-46	-50	-3
S3	-40	-40	28
S4	-52	-29	-15
**Ls-A**			
S1	-45	-46	-7
S2	-49	-57	-5
S3	-41	-58	-2
S4	-44	-57	-6
S5[a]	-31	-77	-6
S6	-44	-51	-9
S6	-36	-55	-8
S8	-47	-31	-15
S9	-43	-37	-12
S10	-44	-32	-12
S11	-42	-56	-4
S12	-47	-38	-11
S13	-40	-33	-14
S14	-43	-52	-2
S15	-44	-35	-11
S16	-46	-61	-12
**Pu-A**			
S1	-40	-45	4
S2[b]	-57	-67	-9
S3[b]	-38	-67	-8
S4a	-39	-84	-14
S5	-44	-32	-14
S6	-44	-59	-11
S7	-39	-49	-15

Ph-A: phonological alexia, Ls-A: lexical-semantic alexia, Pu-A: Pure alexia

[a] Ls-A-S5 and Pu-A-S4 were recorded in the same patient

[b] Pu-A-S2 and Pu-A-S3 were recorded in the same patient

Supplementary Table 2: MNI coordinates (speech disorders)

Stimulations	X coordinate	Y coordinate	Z coordinate
**Ph-P**			
S1	-48	-47	0
S2	-50	-44	7
S3	-53	-44	-6
S4	-47	-53	-6
S5	-42	-60	12
S6	-52	-40	-15
S7	-45	-53	4
S8	-49	-23	-9
S9	-49	-34	-8
S10	-53	-38	-9
S11	-49	-33	-8
S12	-49	-57	-2
S13	-51	-23	-3
S14	-50	-39	-6
S15	-47	-44	-13
**Se-P**			
S1	-47	-49	8
S2	-46	-35	-7
S3	-43	-37	-8
S4	-39	-41	-10
S5	-44	-41	-6
S6	-33	-72	3
S6	-39	-39	-5
S8	-42	-24	-20
S9	-42	-26	-5
S10	-38	-65	4
S11	-41	-38	-11
S12	-32	-10	-11
S13	-40	-45	-2
S14	-44	-9	-14
S15	-42	-31	-8
S16	-40	-38	2
S17	-40	-22	-7
S18	-44	-37	-11
**An**			
S1	-41	-55	-12
S2	-44	-26	-13
S3	-48	-21	-13
S4	-35	-43	-20
S5	-43	-40	29
S6	-49	-38	-6
S7	-41	-30	-11
S8	-41	-50	4
S9	-40	-26	-9
S10	-47	-29	-20
S11	-44	-44	-15
S12	-40	-32	-10

Ph-P: phonological paraphasia, Se-P: semantic paraphasia, An: anomia

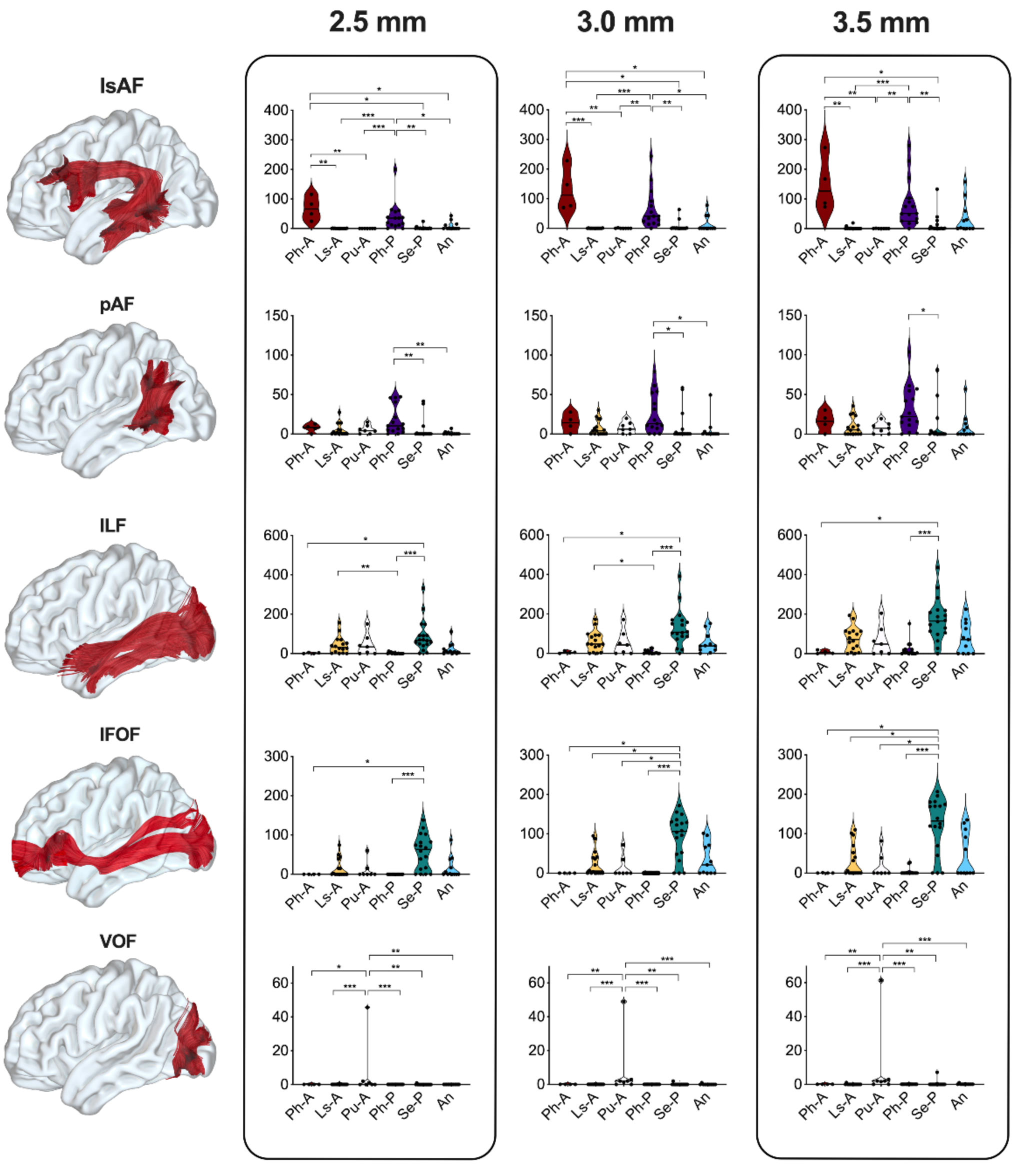

Supplementary figure 2: Tract-level analyses. Distribution of "disconnected" streamlines by VOI size.

Main white matter tracts (lsAF: long segment of the arcuate fasciculus, pAF: posterior segment of the arcuate fasciculus, ILF: inferior longitudinal fasciculus, IFOF: inferior fronto-occipital fasciculus, VOF: vertical occipital fasciculus) according to the HCP-1065 white matter tractography atlas and number of disconnected fibers in each form of alexia (Ph-A: phonological alexia, Ls-A: lexical-semantic alexia, Pu-A: pure alexia) and speech disorders (Ph-P: phonological paraphasia, Se-P: semantic paraphasia, An: anomia).
The total number of generated fibers using 2.5-mm volumes of interest (left), 3.0-mm volumes of interest (middle) and 3.5-mm volumes of interest were collected and projected into the MNI space along with the HCP population-averaged atlas of the human connectome using DSI studio software.
Individual values are presented as dots. Horizontal lines represent the median.
Kruskall-Wallis test and Dunn's test were performed. P-values were adjusted for multiple comparisons.
* $p < 0.05$; ** $p < 0.01$; *** $p < 0.001$

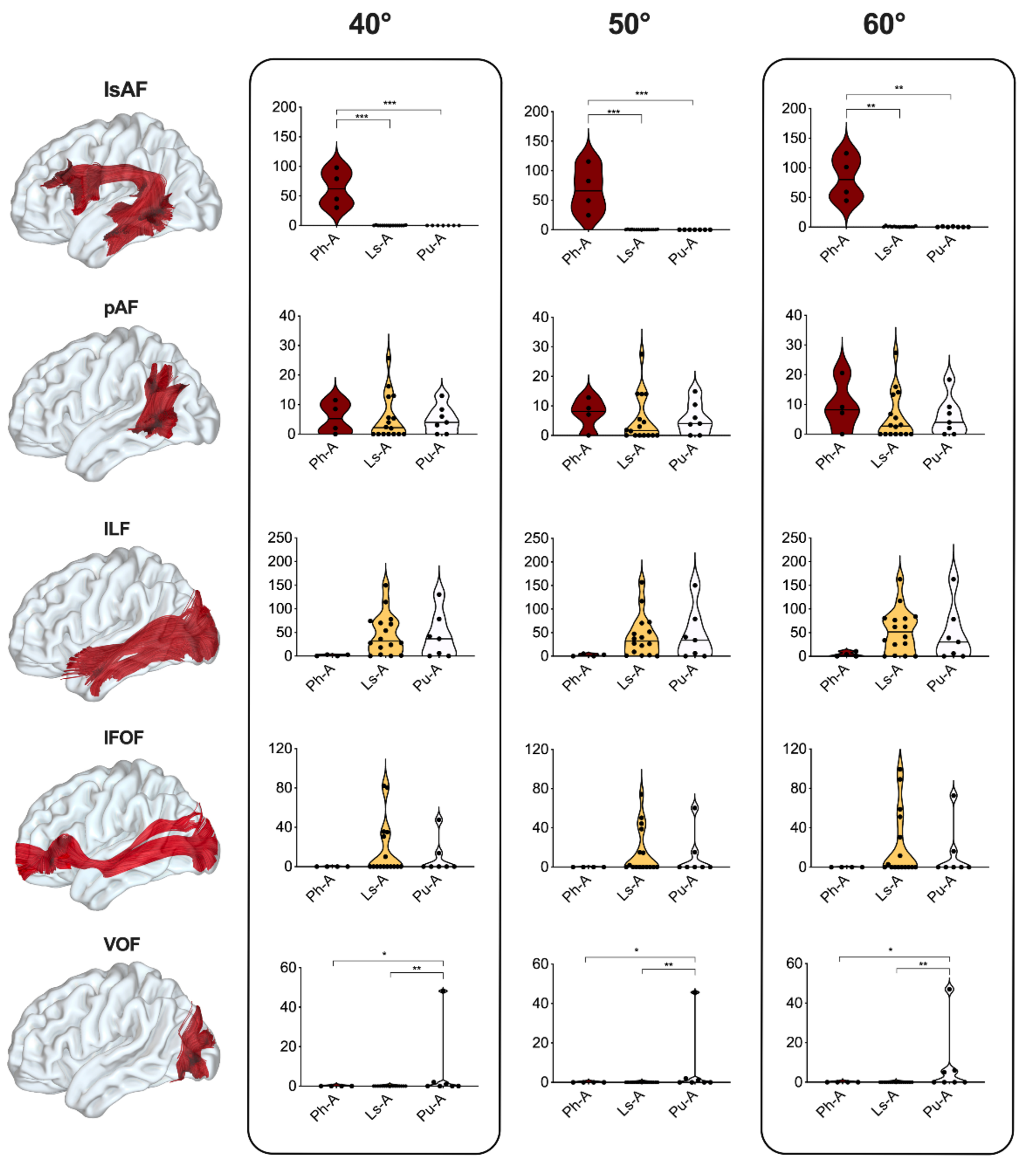

Supplementary figure 3: Tract-level analyses. Distribution of "disconnected" streamlines by angular threshold.

Main white matter tracts (lsAF: long segment of the arcuate fasciculus, pAF: posterior segment of the arcuate fasciculus, ILF: inferior longitudinal fasciculus, IFOF: inferior fronto-occipital fasciculus, VOF: vertical occipital fasciculus) according to the HCP-1065 white matter tractography atlas and number of disconnected fibers in each form of alexia (Ph-A: phonological alexia, Ls-A: lexical-semantic alexia, Pu-A: pure alexia) and speech disorders (Ph-P: phonological paraphasia, Se-P: semantic paraphasia, An: anomia). The total number of generated streamlines using a maximum angular threshold of 40° (left), 50° (middle) and 60°(right) were collected and projected into the MNI space along with the HCP population-averaged atlas of the human connectome using DSI studio software.
Individual values are presented as dots. Horizontal lines represent the median values.
Kruskall-Wallis tests were used to performed group comparisons. P-values were adjusted with the Dunn's test for multiple comparisons. * $p < 0.05$; ** $p < 0.01$; *** $p < 0.001$
