# Supplementary material for: White matter disconnectivity fingerprints causally linked to dissociated forms of alexia
Source: Commun Biol. 2021 Dec 20;4:1413. doi: 10.1038/s42003-021-02943-z (PMC8688436; doi:10.1038/s42003-021-02943-z)
Supplement: Supplementary file 3 — Description of Additional Supplementary Files [file 42003_2021_2943_MOESM3_ESM.pdf]

Description of Additional Supplementary Files

File name: Supplementary Data 1

Description: Summed disconnection matrices in Ph-A (n=4), Ls-A (n=16) and Pu-A (n=7).

File name: Supplementary Data 2

Description: Individual streamline count within the main white matter pathways by alexia subtype.
