# Supplementary material for: White matter disconnectivity fingerprints causally linked to dissociated forms of alexia
Source: Commun Biol. 2021 Dec 20;4:1413. doi: 10.1038/s42003-021-02943-z (PMC8688436; doi:10.1038/s42003-021-02943-z)
Supplement: Supplementary file 6 — Reporting Summary [file 42003_2021_2943_MOESM6_ESM.pdf]

nature portfolio

Corresponding author(s): Sam Ng

Last updated by author(s): Nov 20, 2021

Reporting Summary

Nature Portfolio wishes to improve the reproducibility of the work that we publish. This form provides structure for consistency and transparency in reporting. For further information on Nature Portfolio policies, see our Editorial Policies and the Editorial Policy Checklist.

Statistics

For all statistical analyses, confirm that the following items are present in the figure legend, table legend, main text, or Methods section.

n/a	Confirmed	
☐	☒	The exact sample size (*n*) for each experimental group/condition, given as a discrete number and unit of measurement
☐	☒	A statement on whether measurements were taken from distinct samples or whether the same sample was measured repeatedly
☐	☒	The statistical test(s) used AND whether they are one- or two-sided<br>*Only common tests should be described solely by name; describe more complex techniques in the Methods section.*
☒	☐	A description of all covariates tested
☐	☒	A description of any assumptions or corrections, such as tests of normality and adjustment for multiple comparisons
☐	☒	A full description of the statistical parameters including central tendency (e.g. means) or other basic estimates (e.g. regression coefficient) AND variation (e.g. standard deviation) or associated estimates of uncertainty (e.g. confidence intervals)
☒	☐	For null hypothesis testing, the test statistic (e.g. F, t, r) with confidence intervals, effect sizes, degrees of freedom and P value noted<br>*Give P values as exact values whenever suitable.*
☒	☐	For Bayesian analysis, information on the choice of priors and Markov chain Monte Carlo settings
☒	☐	For hierarchical and complex designs, identification of the appropriate level for tests and full reporting of outcomes
☒	☐	Estimates of effect sizes (e.g. Cohen's *d*, Pearson's *r*), indicating how they were calculated

Our web collection on statistics for biologists contains articles on many of the points above.

Software and code

Policy information about availability of computer code

Data collection	We used intraoperative photography, intraoperative video recordings, clinical records (Dxcare, Epione, Simusante), speech therapist records and neuroimaging data obtained from standard clinical practice. Direct electrostimulation mapping was performed with a Nimbus stimulator (Newmedic, France).
Data analysis	Matlab (Release 2018a, The MathWorksInc, Natick, USA), SPM12, MRIcron software (NITRC, University of Massachussetts, MA, USA) Brain VISA/Abatomist Software (5.0, CEA I2BM, CATI nNeuroimaging, INSERM IFR49 and CNRS, France), Marsbar Toolbox (implemented in Matlab), DSI Studio software (http://dsi-studio.labsolver.org), RStudio 1.3 (R Foudation, for Statistical Computing, Vienna, Austria), Chordiag 0.1.3 package implemented in RStudio.

For manuscripts utilizing custom algorithms or software that are central to the research but not yet described in published literature, software must be made available to editors and reviewers. We strongly encourage code deposition in a community repository (e.g. GitHub). See the Nature Portfolio guidelines for submitting code & software for further information.

Data

Policy information about availability of data

All manuscripts must include a data availability statement. This statement should provide the following information, where applicable:

- Accession codes, unique identifiers, or web links for publicly available datasets
- A description of any restrictions on data availability
- For clinical datasets or third party data, please ensure that the statement adheres to our policy

Data necessary to the reproducibility of the results are provided in the Supplementary files. Other data can be obtained upon request by contacting Dr Sam Ng (s-ng@chu-montpellier.fr)

Field-specific reporting

Please select the one below that is the best fit for your research. If you are not sure, read the appropriate sections before making your selection.

- [x] Life sciences
- [ ] Behavioural & social sciences
- [ ] Ecological, evolutionary & environmental sciences

For a reference copy of the document with all sections, see nature.com/documents/nr-reporting-summary-flat.pdf

Life sciences study design

All studies must disclose on these points even when the disclosure is negative.

Sample size	No sample size calculation was performed
Data exclusions	N/A
Replication	All electrostimulation applications inducing an intraoperative disturbance were replicated at least 3 times. Stimulations inducing reading/ language disorders at least 3 times on the same subcortical/cortical site were selected for analysis.
Randomization	N/A
Blinding	All intraoperative neurocognitive/ reading disorders were collected by a senior speech therapist who remained blinded to electrostimulation application.

Reporting for specific materials, systems and methods

We require information from authors about some types of materials, experimental systems and methods used in many studies. Here, indicate whether each material, system or method listed is relevant to your study. If you are not sure if a list item applies to your research, read the appropriate section before selecting a response.

Materials & experimental systems

n/a	Involved in the study	
x		Antibodies
x		Eukaryotic cell lines
x		Palaeontology and archaeology
x		Animals and other organisms
x		Human research participants
	x	Clinical data
x		Dual use research of concern

Methods

n/a	Involved in the study	
x		ChIP-seq
x		Flow cytometry
	x	MRI-based neuroimaging

Clinical data

Policy information about clinical studies

All manuscripts should comply with the ICMJEguidelines for publication of clinical research and a completedCONSORT checklist must be included with all submissions.

Clinical trial registration	Approval for this study was granted by the Institutional Review Board of Montpellier University Center (N°202000557)
Study protocol	The study protocol is provided in the manuscript and was previously published in cited papers.
Data collection	All data were obtained from a clinical context without additional intervention.
Outcomes	Electrostimulation mapping findings, based on an asleep-awake-asleep protocol during brain tumor resection, i.e. intraoperative language disorders (either verbal and non-verbal disorders), reading disorders, visuospatial disorders, mentalizing disorders or self-evaluation disorders.

Magnetic resonance imaging

Experimental design

Design type	Standard structural neuroimaging (FLAIR, T1, T sequences)
Design specifications	NA

Behavioral performance measures	Neuroimaging data were confronted to intraoperative behavioral findings.

Acquisition

Imaging type(s)	Structural
Field strength	3T
Sequence & imaging parameters	T1,T1 with Gadolinium, T2, FLAIR either preoperatively and 3 months after surgery
Area of acquisition	Whole brain

Diffusion MRI ☒ Used ☐ Not used

Parameters: Diffusion data (population-averaged streamlines) from the Human Connectome Project were used.

Preprocessing

Preprocessing software	Diffusion data from the HCP were used (pre-processing steps are fully detailed in cited publications)
Normalization	Structural data were normalized using SPM12, implemented in the MATLAB environnment.
Normalization template	N/A
Noise and artifact removal	N/A
Volume censoring	N/A

Statistical modeling & inference

Model type and settings	N/A
Effect(s) tested	N/A

Specify type of analysis: ☐ Whole brain ☒ ROI-based ☐ Both

Anatomical location(s): Stimulation points were used as ROIs to generate streamline disconnection and disconnection matrices in DSI studio (HCP datasets).

Statistic type for inference (See Eklund et al. 2016)	N/A
Correction	FDR

Models & analysis

n/a	Involved in the study	
☒	☐	Functional and/or effective connectivity
☒	☐	Graph analysis
☒	☐	Multivariate modeling or predictive analysis
